# Differentially Private and Skew-Aware Spatial Decompositions for Mobile Crowdsensing

**DOI:** 10.3390/s18113696

**Published:** 2018-10-30

**Authors:** Jong Seon Kim, Yon Dohn Chung, Jong Wook Kim

**Affiliations:** 1 Department of Computer Science and Engineering, Korea University, Seoul 02841, Korea; jongseon9312@gmail.com (J.S.K.); ydchung@korea.ac.kr (Y.D.C.); 2 Department of Computer Science, Sangmyung University, Seoul 03016, Korea

**Keywords:** spatial databases, differential privacy, histograms, mobile crowdsensing

## Abstract

Mobile Crowdsensing (MCS) is a paradigm for collecting large-scale sensor data by leveraging mobile devices equipped with small and low-powered sensors. MCS has recently received considerable attention from diverse fields, because it can reduce the cost incurred in the process of collecting a large amount of sensor data. However, in the task assignment process in MCS, to allocate the requested tasks efficiently, the workers need to send their specific location to the requester, which can raise serious location privacy issues. In this paper, we focus on the methods for publishing differentially a private spatial histogram to guarantee the location privacy of the workers. The private spatial histogram is a sanitized spatial index where each node represents the sub-regions and contains the noisy counts of the objects in each sub-region. With the sanitized spatial histograms, it is possible to estimate approximately the number of workers in the arbitrary area, while preserving their location privacy. However, the existing methods have given little concern to the domain size of the input dataset, leading to the low estimation accuracy. This paper proposes a partitioning technique SAGA (Skew-Aware Grid pArtitioning) based on the hotspots, which is more appropriate to adjust the domain size of the dataset. Further, to optimize the overall errors, we lay a uniform grid in each hotspot. Experimental results on four real-world datasets show that our method provides an enhanced query accuracy compared to the existing methods.

## 1. Introduction

With the rapid developments of information technology, we are using various mobile devices during daily activity. Mobile devices, equipped with several sensors, enable users to measure and monitor environmental conditions such as air pollution, temperature and humidity. Accordingly, a paradigm to collect large-scale sensing data through the prevalent mobile devices has recently emerged, which is referred to as Mobile Crowdsensing (MCS). With MCS, the requesters assign the set of tasks to the participating workers, and the workers move to specific locations and perform the assigned tasks. Typically, this process is performed by the MCS server, which plays a role as the intermediary [1].

For example, the air pollution problem in smart cities is one of the issues that requires large-scale sensing data [2]. However, it is very costly to maintain numerous sensors in urban areas. Here, MCS can be an alternative way to monitor the air quality at a low cost. Consider a scenario where each worker holds a mobile device that can monitor current air quality conditions. A requester wants to collect air quality data in a particular region and transmits the tasks of sensing air quality to the MCS server. Then, the MCS server assigns the tasks to the workers who exist in the areas of concern. Specifically, the MCS server needs an exact spatial distribution of workers, because the workers would not perform the tasks if the travel distance is too long. In other words, for efficient task assignment, the workers need to send their precise location to the MCS server, which can raise serious privacy concerns, because the exact locations of workers can be leaked to an attacker. Thus, ensuring the location privacy of workers is an important issue, because the workers would not engage in MCS if their location privacy were not appropriately protected.

The location data often include person-specific information and can reveal considerable details about the individual’s life. For example, an adversary can track or monitor a particular individual based on the location data [3]. Accordingly, the adversary can infer habits, social customs, religious beliefs and sexual preferences of individuals based on their mobility traces, which can lead to privacy breaches. Even worse, it has been shown that simple anonymization (just removing the obvious identifiers) cannot completely prevent location privacy attacks. In summary, we need a stronger location privacy-preserving method when collecting the sensor data from the workers.

Differential Privacy (DP) [4] is the de facto standard model for privacy-preserving data analysis. DP provides strong privacy protection by randomizing the analysis results. Although anonymization-based privacy models such as *k*-anonymity [5], *l*-diversity [6] and *t*-closeness [7] cannot ensure privacy from adversaries with background knowledge more than the *k*, *l*, *t* values  [8], DP can provide a rigorous privacy guarantee against an adversary with arbitrary background knowledge. With the notion of differential privacy, a spatial histogram publishing technique has been introduced under the name of Private Spatial Decomposition (PSD) [9].

PSD takes the form of spatial indexes such as kd-trees, quadtrees and grids [9,10,11,12,13]. These methods partition a spatial domain into several regions and add carefully calibrated noise to the count of objects within the boundaries of each region. Following this, PSD can be directly used to estimate the number of objects in arbitrary range queries.

**Figure 1:**
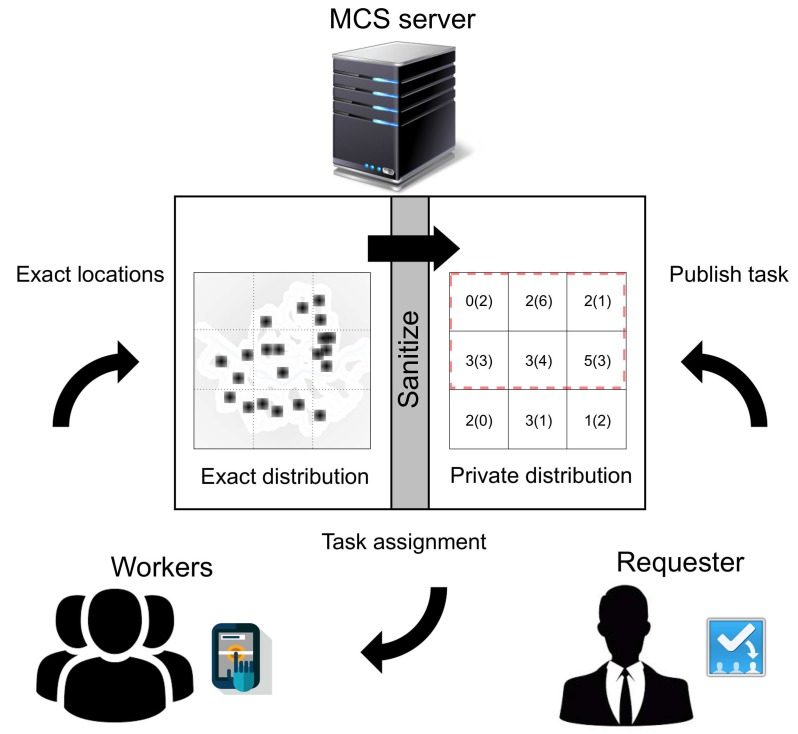
A framework for mobile crowdsensing using PSD. MCS, Mobile Crowdsensing.

**Example** **1.** *(Selectivity estimation during mobile crowdsensing using PSD) Figure 1 represents a framework for the private task assignment process. In this paper, we assume the following situation. First, the workers submit their exact locations to the trusted MCS server. Then, the MCS server builds a PSD, which provides the private distribution of workers. After that, the untrusted requesters assign the tasks based on the query results from the PSD. The requesters can estimate the number of people in a certain range based on the PSD. Here, the left/right numbers of PSD represent the actual/noisy number of workers in each divided region. Let us consider a range query that is represented by a dotted rectangle on the PSD. It includes three top and middle grid cells. The result of the range query can be estimated as (2 + 6 + 1 + 3 + 4 + 3) = 19.*

Our objective is to design a PSD method that provides an accurate representation of worker distribution while protecting the location privacy of the workers during the task assignment process. Existing PSD approaches can be classified into two categories: data-independent [11,13,14] and data-dependent methods [9,10,12]. Data-independent methods partition the spatial domain without considering the distribution of data. For this reason, when the data distribution is highly skewed, these methods create many meaningless zero-count cells. In contrast, data-dependent methods partition the spatial domain based on the distribution of data. To determine the data distribution fully, these methods require many levels. However, because the privacy budget is limited, each level might use a small privacy budget, resulting in higher estimation errors.

Moreover, most of the PSD methods do not consider the domain size of the input dataset, except the work in [14]. The work in [14] optimized the grid size based on the linear regression analysis. However, this process needs several sample query results, leading to potential privacy leaks and overfitting their granularity to the input dataset. To solve this problem, we propose a novel PSD method, called SAGA (Skew-Aware Grid pArtitioning). Our method first detects the hotspots, which have a higher density than that of the entire spatial domain. This process divides the large domain into multiple smaller sub-regions, which can handle errors more locally. Subsequently, SAGA lays a uniform grid in each hotspot to optimize the overall errors. Through extensive experiments, we compare our method to the state-of-the-art methods and demonstrate that SAGA achieves better range query estimation accuracy.

In MCS, we have to consider both the location privacy of the workers and the optimal allocation of tasks. However, it is challenging to estimate the number of workers in a region accurately given a PSD, because the exact number of workers should not be released. Thus, it is an important issue to provide an accurate noisy count to improve the rates of task acceptance. We found that the domain size and density of an input dataset are two major factors affecting the accuracy of PSD. The existing PSD methods assume that the workers are uniformly distributed in each sub-region. However, in the real world, the workers are unevenly distributed and tend to gather with each other, forming a cluster. Thus, the uniform distribution assumption only holds when the domain size is small. This insight gives us an idea of utilizing hotspots.

To summarize, we make the following contributions in this paper:

- We propose a novel PSD method called SAGA. The proposed SAGA constructs a histogram based on hotspots, which are the regions with high density. This process effectively adjusts the domain size of input datasets. SAGA then adaptively lays a uniform grid in each hotspot, optimizing the total estimation error.
- We formally prove that the proposed SAGA satisfies ε-differential privacy. We also present the heuristics that privately choose the required parameter values of SAGA.
- We conduct extensive experiments using four real datasets. The experimental results demonstrate that SAGA achieves better utility than the existing state-of-the-art techniques.

The remainder of this paper is organized as follows. Section 2 describes related work. In Section 3, we introduce PSD with some necessary background knowledge. Section 4 presents the skewness problem and our approach. Experimental results and the discussion are provided in Section 5. Finally, in Section 6, we conclude this paper.

## 2. Related Work

In this section, we present several studies related to the privacy challenges in mobile crowdsensing.

### 2.1. Location Privacy in Mobile Crowdsensing

Mobile crowdsensing (MCS) was coined by Ganti et al. [1], which refers to a technique where individuals utilize their sensing devices to collect information about their environment. MCS has recently drawn considerable attention due to its power of large-scale sensing. Specifically, the MCS process has four phases: task creation, task assignment, individual task execution and crowd data integration [15]. The MCS server needs the specific location of the workers during the task assignment process, and the workers have a risk of location exposure. Accordingly, there have been several studies that guarantee privacy during the task assignment process [16,17,18].

There were already large research studies associated with location privacy-preserving techniques, which are adopted in the MCS [19]. Specifically, *k*-anonymity-based solutions [20,21,22] have been proposed, which make at least *k* users share the same region. Different from the *k*-anonymity-based methods, differential privacy [23,24] is now widely adopted, which ensures a strong privacy guarantee. There are also considerable studies that adopt differential privacy to sanitize spatial and spatiotemporal data [9,10,11,12,13,25,26].

### 2.2. Data-Dependent PSD

Xiao et al. [10] proposed a method of releasing a multi-dimensional histogram using a kd-tree. A kd-tree begins from a node that covers the entire space. This node is then recursively binary-partitioned along a chosen dimension. To decrease the non-uniformity error, they proposed a uniformity heuristic. This heuristic splits a node if the resulting two sub-regions nearly have the same number of points.

Cormode et al. [9] proposed several alternative kd-tree heuristics. Specifically, they split kd-tree nodes based on the median points with respect to the chosen dimension. In addition, they proposed a hybrid method that generates a kd-tree for the first few levels and splits the sub-regions into four equal quadrants for the remaining levels.

To et al. [12] proposed an h-tree-based PSD. h-tree is an equi-depth histogram that provides a similar number of points in each cell. Because it uses only two levels, each level of h-tree could use a greater privacy budget than each level of kd-tree. In addition, to reduce the number of splits, h-tree splits each dimension based on the median point. In other words, to create *m* number of nodes, the naive split approach requires m−1 splits, whereas the median split approach needs only $\left\lceil \log_{2}m\right\rceil$ splits. It is known that h-tree provides better query accuracy than kd-tree and grid-based PSD methods when the distribution is skewed [12].

### 2.3. Data-Independent PSD

Quardaji et al. [11] proposed a uniform grid method. Specifically, they proved that a grid size $m=\sqrt{\frac{N\varepsilon }{c}}$ minimizes the sum of perturbation and non-uniformity errors (*N* is the total number of data points in a dataset and *c* is a constant). They demonstrated that a uniform grid PSD with the above grid size tends to have better query accuracy than the kd-tree-based PSD methods. They also proposed an adaptive grid method that lays another uniform grid on each first level grid cell based on the noisy count. Accordingly, the adaptive grid method possesses a fine granularity grid over the dense region, while having a coarse grid over the sparse region.

Zhang et al. [13] proposed a quadtree-based PSD known as PrivTree. While the traditional quadtree-based methods divide each cell until reaching a predefined tree height, PrivTree splits each node based on the biased count. To do this, they defined a threshold that limits the height of a quadtree. Because the privacy budget is not used to determine the biased count, the amount of noise added to the PrivTree is constant regardless of the height of PrivTree.

Wang et al. [14] proposed a novel granularity grid-based PSD. They focused on the problem that when the partially overlapping region between the grid cells and the range query becomes bigger, the non-uniformity error also increases. They exploited several query results to infer the relationship between the overlapping area and non-uniformity error through linear regression analysis. They also proposed a method of merging similar grid cells, which further reduces the noise added to each cell. However, their strategy can potentially leak information about the dataset, since the granularity depends on the results from the original data [27].

## 3. Preliminaries

In this section, we introduce the notion of differential privacy and the mechanisms for achieving differential privacy, followed by the Private Spatial Decomposition (PSD).

### 3.1. Differential Privacy

Differential privacy requires that the outputs of any query should be approximately the same whether a single record is added or removed from the database. Formally, differential privacy is defined as follows.

**Definition** **1.** *(ε-differential privacy) Assume a mechanism A that randomizes the query outputs and any pair of neighboring databases D and $D^{\prime }$. Then, A satisfies ε-differential privacy if and only if:*

(1) $$Pr\left[A\left(D\right)=S\right]\leq exp\left(\varepsilon \right)\times Pr\left[A\left(D^{\prime }\right)=S\right]$$

*where S∈Range(A).*

In this paper, we consider two databases D and $D^{\prime }$, which are neighboring if and only if they differ in only one record. In other words, we can obtain $D^{\prime }$ from D by removing or adding an arbitrary record. If a mechanism satisfies Equation 1, D and $D^{{}^{\prime }}$ have a very high probability of outputting the same results. Accordingly, an adversary with arbitrary background knowledge cannot infer a particular record.

**Definition** **2.** *(Sensitivity) Given any two databases D and $D^{\prime }$, the sensitivity of a query F is defined as:*

(2) $$\Delta F=\max_{D,D^{\prime }}\left|F\left(D\right)-F\left(D^{\prime }\right)\right|$$

*where D and $D^{\prime }$ are neighboring.*

Sensitivity refers to the extent to which an arbitrary record maximally affects the query results. For example, the sensitivity of count query is one because a particular record may or may not satisfy the query predicate. Based on the sensitivity of a query, a mechanism randomizes the query results to achieve differential privacy.

There are typically two mechanisms that can achieve differential privacy: the Laplace [23] and exponential [28,29] mechanism. The Laplace mechanism is a random real value adding method.

**Theorem** **1.** *(Laplace mechanism) Let F(D) denote a query result from database D. The Laplace mechanism satisfies ε-differential privacy if a random noise sampled from a Laplace distribution with mean μ=0 and scale b=ΔF/ε is added to F(D).*

**Proof.**  Refer to [23]. □

The second mechanism is the exponential mechanism [28,29]. The exponential mechanism is used when adding real values to query results makes no sense. This mechanism first assigns a score to possible query results using a score function. An output with a higher score indicates that it is closer to the true output. The mechanism then randomly selects an output from the possible query result set; the higher the score, the more appealing the result.

**Theorem** **2.** *(Exponential mechanism) Let R be the possible results of a query F. For a score function S:D×R→R, a mechanism that outputs r∈R with a probability that is proportional to exp(εS(D,r)/2ΔS) satisfies ε-differential privacy, where ΔS is the sensitivity of S.*

**Proof.**  Refer to [28]. □

Differentially private mechanisms are composable [29]. In other words, if an algorithm consists of multiple differentially private mechanisms, then the algorithm also satisfies the differential privacy requirement.

**Theorem** **3.** *(Sequential composition) Suppose an algorithm F consists of $\varepsilon_{i}$-differential privacy mechanisms running on the same dataset. Then, F provides $\left(\sum_{i}\varepsilon_{i}\right)$-differential privacy.*

**Proof.**  Refer to [29]. □

**Theorem** **4.** *(Parallel composition) Suppose an algorithm F consists of $\varepsilon_{i}$-differential privacy mechanisms running on the disjoint dataset. Then, F provides $\max_{i}\left(\varepsilon_{i}\right)$-differential privacy.*

**Proof.**  Refer to [29]. □

Because of the composition theorems, we usually refer to ε as the privacy budget [23]. To satisfy the ε-differential privacy requirement, each part of an algorithm uses a portion of ε, and the sum of privacy budget should be no more than ε.

### 3.2. Private Spatial Decomposition

The objective of this paper is to release a differentially private two-dimensional spatial histogram that provides an accurate range count query estimation. Given a spatial database, we consider each record as a point in the two-dimensional spatial domain. PSD methods first partition the domain into several cells and then add noise to each cell in a way that satisfies differential privacy. In summary, PSD consists of the boundary of these cells along with the noisy counts, as shown in Figure 1. This PSD can then be used directly to answer arbitrary range count queries.

When estimating the count using PSD, two sources of errors can arise: the perturbation and non-uniformity error. Next, we explain these two errors in detail with examples.

**Figure 2:**
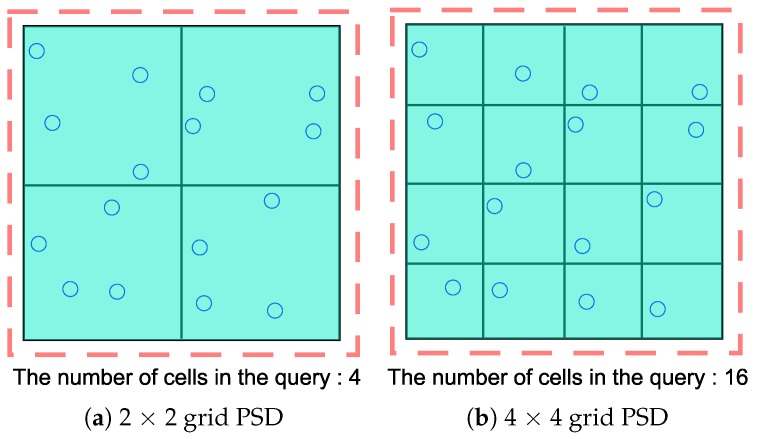
Two different grid PSDs in the same spatial domain.

**Example** **2.** *(Perturbation error) Figure 2 presents examples of different space decompositions in the same spatial domain, which includes 16 location points. To satisfy ε-differential privacy, we should add an independently generated noise to each cell count. These noises are sampled from the Laplace distribution with a variance $\sigma =2{\left(\Delta F/\varepsilon \right)}^{2}$. Because these noises are independently generated, the variance of perturbation error is proportional to the number of cells included in a query. Consider a range query that includes the entire spatial domain. PSD of Figure 2a will give a result with the error variance of 4σ, whereas the error variance of Figure 2b is 16σ. Therefore, the greater the number of cells that are contained in a query and the finer the granularity of PSD, the larger will be the perturbation error.*

**Figure 3:**
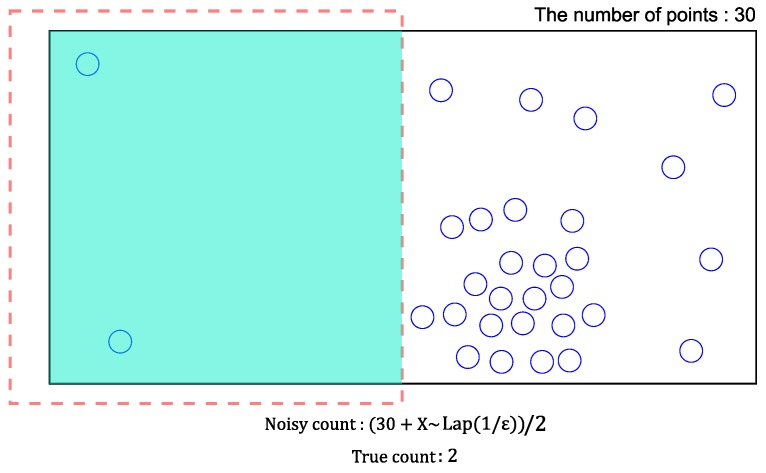
A PSD cell that contains points with a skewed distribution.

**Example** **3.** *(Non-uniformity error) In Figure 3, most of the points exist in the right part of this cell. Consider a range query represented by a dotted rectangle. Because one-half of the cell overlaps the query, this cell will give the estimated count of $\frac{\left(30+X∼Lap(1/\varepsilon )\right)}{2}$ based on the uniform distribution assumption. However, this value significantly differs from the real count of two. In other words, the non-uniformity error becomes larger when the distribution of points in a cell are not uniform.*

All the cells in the query induce the perturbation error because of the Laplace noise we added in each cell, while only the cells that partially intersect with the query contribute to non-uniformity error. As the size of range query increases, the non-uniformity error tends to decrease because most of the cells are fully included. On the other hand, the perturbation error grows because the number of cells included increases. Therefore, it is important to balance this trade-off.

## 4. The Proposed Method: SAGA

In this section, we first identify the skewness problem that existing PSD methods suffer from when the distribution of data is skewed. We then present our SAGA (Skew-Aware Grid pArtitioning) approach that overcomes the problem.

### 4.1. The Skewness Problem

**Figure 4:**
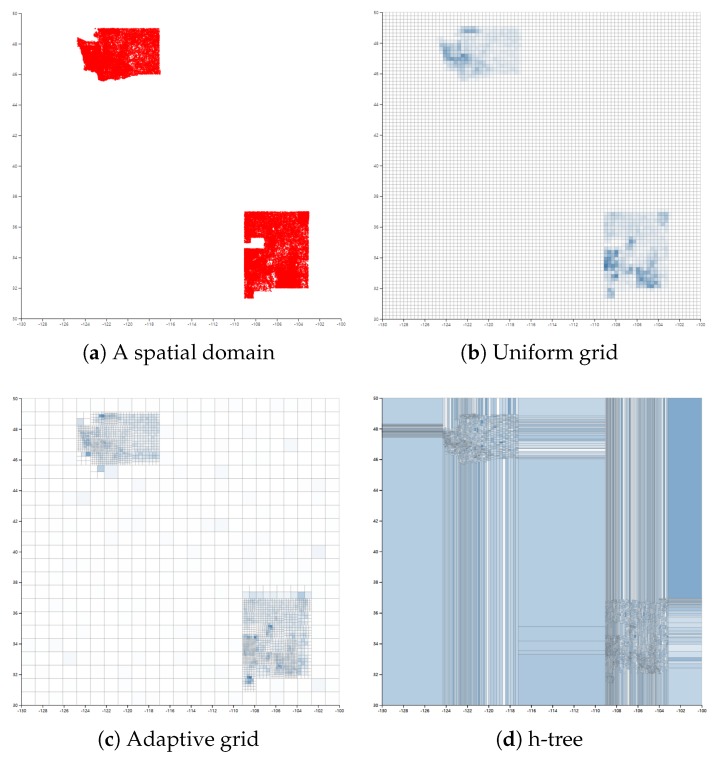
Examples of a spatial domain and various corresponding PSD structures.

Existing PSD methods have some problems when the data distribution is skewed. Figure 4a represents an example of a spatial distribution where most of the data points are clustered in the top-left and bottom-right corner. The corresponding histogram structures of the uniform grid, adaptive grid [11] and h-tree [12] are illustrated in Figure 4b–d.

PSD based on the grid methods considers all regions identically. Consequently, these methods create many zero-count cells in the sparse regions and insufficient cells in the dense regions, which incur high perturbation error and non-uniformity error, respectively. Adaptive grid overcomes this problem by first laying a coarse $m_{1}\times m_{1}$ grid and then partitioning each first level cell into an $m_{2}\times m_{2}$ grid based on the noisy count of each first level cell. Despite the hierarchical structure, the adaptive grid still suffers from the same problem as the uniform grid does.

h-tree attempts to overcome this problem by adopting a two-level equi-depth histogram, which provides a similar number of points in each cell. Assuming that each cell has a similar number of points, the probability of being non-uniform is relatively high for the large cells. However, since the h-tree does not take into account the size of cells, the size of cells in the sparse region is relatively large. Though h-tree provides enhanced range query accuracy compared to the grid-based PSD methods on the skewed dataset, h-tree still does not show better performance on the relatively uniform dataset [12].

Other existing density-based PSD methods also try to handle the skewness problem by partitioning the region recursively and constructing a hierarchical histogram. However, these methods do not consider the domain size of input datasets. Each divided sub-region receives the independent Laplace noises to guarantee differential privacy. Therefore, when answering the range query, the perturbation error increases linearly with the number of sub-regions included in this range query. In other words, for a large range query, the perturbation error is higher than the non-uniformity error. On the other hand, when the small range query is applied, the portion of the non-uniformity error within the estimation error is higher than that of the perturbation error. Accordingly, the utility of the PSD critically depends on the domain size of the input datasets.

SAGA tries to adjust the domain size by first detecting a region where a collection of objects is densely grouped. Then, by laying a uniform grid in these regions, our method balances the perturbation and non-uniformity error. Thus, SAGA demonstrates consistent estimation accuracy regardless of the properties of input datasets.

### 4.2. The SAGA Approach

In this section, we develop a privacy budget allocation strategy for SAGA histogram construction to achieve differential privacy. We adopt the technique developed by Roh [30] to handle the skewness problem efficiently. In the study of [30], the histogram consists of the dense sub-regions called hotspots. We first introduce the notion of hotspots. We then discuss the ways of privately detecting the hotspots and generating the uniform grid in the hotspots.

The construction of SAGA histograms consists of two phases: (1) the entire spatial domain is first partitioned based on the hotspots; (2) each hotspot is further split into the uniform grid. Accordingly, to achieve ε-differential privacy, the total privacy budget ε should be divided into two phases, $\varepsilon^{b}+\varepsilon^{c}=\varepsilon$. Each privacy budget $\varepsilon^{b}$ and $\varepsilon^{c}$ is respectively used to decide the boundary of hotspots and the Laplace noise added to the actual count value.

#### 4.2.1. Notion of Hotspots

Hotspot indicates a sub-region that has a high relative density. In this paper, the density is calculated as $\frac{Freq(R)}{Size(R)}$. Size(R) denotes the size of the region *R*, and Freq(R) denotes the number of data points in *R*. To define a hotspot, we use two parameters, *s* and *f*. Each parameter decides the size and the minimum number of points in hotspots and consequently determines the density of hotspots.

**Definition** **3.** *(Hotspot) Suppose the size of super-region $R_{sup}$ is S and the number of points in $R_{sup}$ is F. Given two parameters s and f, a sub-region $R_{sub}$ can be a hotspot if the following conditions are satisfied.*

*(1)*  *Size condition: $Size\left(R_{sub}\right)\leq \frac{S}{s}$.*
*(2)*  *Frequency condition: $Freq\left(R_{sub}\right)\geq \frac{F}{f}$.*
*(3)*  *Exclusiveness condition: Any two hotspots are mutually exclusive.*

**Figure 5:**
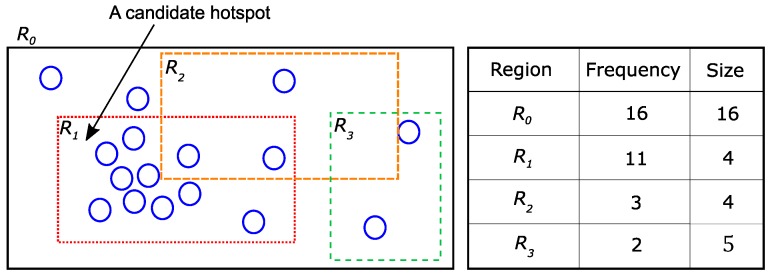
An example of the hotspot.

**Example** **4.** *(Hotspot) Figure 5 shows a super-region $R_{0}$ and three sub-regions $R_{1},R_{2}$ and $R_{3}$. The size and frequency of each region are described in the right table of Figure 5. Suppose the given parameter values are s=4 and f=2. In other words, the density of the hotspot should be at least $\frac{s}{f}=2$-times larger than that of its super-region. Sub-region $R_{1}$ satisfies all conditions ($Size\left(R_{1}\right)=4\leq \frac{16}{4}$, $Freq\left(R_{1}\right)=11\geq \frac{16}{2}$). Sub-region $R_{2}$ satisfies the size condition ($Size\left(R_{2}\right)=4\leq \frac{16}{4}$), but does not satisfy the frequency condition ($Freq\left(R_{2}\right)=3<\frac{16}{2})$. Sub-region $R_{3}$ does not satisfy any conditions.*

#### 4.2.2. Hotspots Detection

In this section, we discuss the ways of using the privacy budget $\varepsilon^{b}$ to privately decide the hotspot boundaries. We present the full hotspot detection algorithm. We first briefly describe the hotspot detection algorithm. We detect the hotspots by using the sliding window mechanism. The reason is that searching all the sub-regions that satisfy the hotspot conditions is computationally infeasible. Therefore, we use a basic rectangle with the size of $\frac{S}{s}$ to efficiently detect the hotspots. This rectangle’s width (height) is $\frac{1}{\sqrt{s}}$ times the width (height) of the entire spatial domain. Although this is not the optimal shape, the boundaries of the detected hotspots are privately adjusted later.

**Figure 6:**
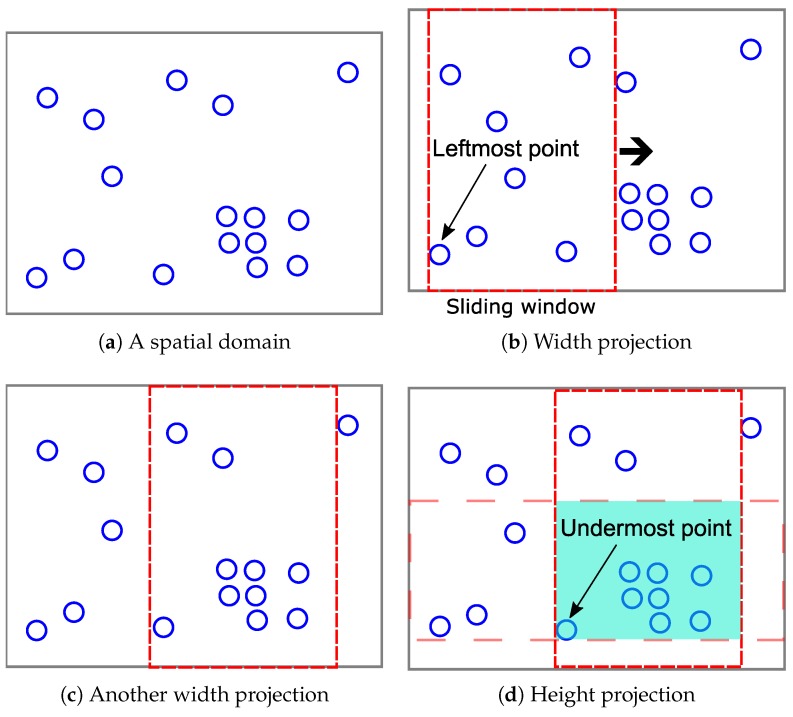
Hotspot detection using the sliding window mechanism.

Suppose two parameters are given as s=4 and f=2 and there is a super-region (the entire space) depicted in Figure 6a. There are 16 points in this space. First, a sliding window starts from the X-axis coordinate of a leftmost point. Then, the algorithm checks the frequency condition: whether the number of points in this sliding window is greater than or equal to $\frac{F}{f}$. In Figure 6b, the number of points is seven, and so, the sliding window starts from the next points. In the case of Figure 6c, the number of points is 10, and so, now, another sliding window moves from the Y-axis coordinate of an undermost point. In Figure 6d, because the number of points in the overlapping region is eight, this region is considered a hotspot.    
**Algorithm 1***BoundaryGeneration* procedure.**Require:** a sorted coordinate value list *V*, boundary privacy budget $\varepsilon^{\tilde{b}}$ **Ensure:** a noisy boundary coordinate value *v*
1:**for**i=0 to |V|−2 **do**2:    $I_{i}=\left[V\left[i\right],V\left[i+1\right]\right)$ ← a coordinate interval value3:    $rank(I_{i})=i$4:**end for**5:Choose an interval $I_{k}$ with probability ∝$|I_{k}|\times exp\left(-\varepsilon^{\tilde{b}}\times rank\left(I_{k}\right)/2\right)$6:Choose a random value *v* from $I_{k}$7:**return***v*


**Figure 7:**
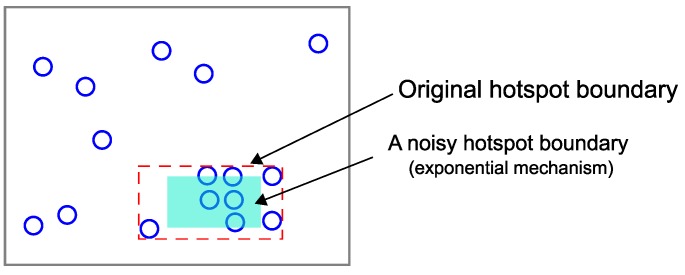
A noisy hotspot boundary of Figure 6d by Algorithm 1.

As explained above, the hotspot detection process is performed based on the real coordinates of points. In other words, SAGA is a data-dependent PSD. Accordingly, simply adding the Laplace noise to the actual count value of each hotspot is not enough to guarantee the given differential privacy requirement: an adversary could infer a particular location point from the structure of the hotspots. Therefore, a hotspot boundary (bottom-left and top-right coordinates) should be formulated in a differentially-private way. Figure 7 describes an example of a noisy hotspot boundary decided by Algorithm 1.

Algorithm 1 uses the exponential mechanism to sample the values of random coordinates. We explain here that there are two reasons for using the exponential mechanism: (1) adding Laplace noises directly to the real coordinate values can cause a breach in the exclusiveness condition; and (2) directly drawing a boundary from the set of values has high sensitivity. Suppose the real coordinates value is $x_{h}$ and Laplace noise is X∼Lap(1/ε). Then, the noisy coordinate value is $x_{h}+X$. However, because the Laplace noise is an arbitrary real value, a hotspot can deviate from the domain of the input dataset and overlap. In addition, directly drawing the boundary from the set of points in a hotspot has high sensitivity, resulting in a larger boundary and estimation error [31].

In Algorithm 1, we use the rank() function as the score function of the exponential mechanism. Algorithm 1 first gives a rank score to an interval $I_{i}$ in ascending order (Line 3). This means that if an interval is close to the original hotspot boundary, this interval receives a lower score (the lower the score, the higher the rank), which in turn has a higher chance of being chosen. Then, Algorithm 1 randomly draws an interval with a probability that is proportional to the rank score (Line 5). Because all coordinate values in $I_{k}$ have an equal rank score, a noisy coordinate value is uniformly chosen at random, and this value becomes the noisy hotspot coordinate (Line 6).

**Theorem** **5.** *Algorithm 1 achieves $\varepsilon^{\tilde{b}}$-differential privacy.*

**Proof.**  In Algorithm 1, we use the rank() function as a score function S of the exponential mechanism. Because adding or removing a value in the coordinate list changes the rank of any coordinate value by at most one, the sensitivity of the rank() function is one (ΔS=1). In the determination of the left boundary, Algorithm 1 assigns the leftmost interval $I_{0}=\left[x_{0},x_{1}\right)$ to the rank score of zero, the second leftmost interval $I_{1}=\left[x_{1},x_{2}\right)$ to the rank score of one, and so on. According to Theorem 2, if Algorithm 1 chooses an interval $I_{k}=\left[p_{k},p_{k+1}\right)$ with probability $\frac{|I_{k}|exp\left(-\varepsilon^{\tilde{b}}\times rank\left(I_{k}\right)/2\right)}{\sum_{i=0}^{i=n-1}\left|I_{i}\right|exp\left(-\varepsilon^{\tilde{b}}\times rank\left(I_{i}\right)/2\right)}$, where n is the size of the coordinate value list, then Algorithm 1 satisfies the $\varepsilon^{\tilde{b}}$-differential privacy. After the interval $I_{k}$ is selected, Algorithm 1 returns a single uniformly-distributed random coordinate value from the chosen interval $I_{k}$, which does not affect the privacy guarantee of the exponential mechanism. □

**Theorem** **6.** *Hotspot boundary generation achieves $\varepsilon^{b}$-differential privacy.*

**Proof.**  To formulate a noisy hotspot boundary, we need four coordinate values (bottom-left and top-right). Accordingly, the BoundaryGeneration procedure (Algorithm 1) is applied to the same point set four times. By Theorem 3, we should divide the privacy budget $\varepsilon^{b}$ by four $\left(\varepsilon^{b}/4\right)$. Moreover, because each hotspot contains a disjoint set of location points, by the exclusiveness condition, according to Theorem 4, we can use the entire budget $\varepsilon^{b}$ to decide the noisy boundary of each hotspot. □

#### 4.2.3. Grid Partitioning and Parameter Value Choice

In this section, we explain how the other privacy budget $\varepsilon^{c}$ is used and the choice of parameter values. After detecting the hotspots and formulating the noisy boundaries using the privacy budget $\varepsilon^{b}$, the other privacy budget $\varepsilon^{c}$ is used to add Laplace noise to the actual count value of each hotspot. Though the original hotspot boundaries are almost minimum bounding rectangles that contain high-density point clusters, the distribution can still be skewed because of the randomness of the exponential mechanism (Figure 7). Accordingly, the accuracy of range query estimation can be reduced.

Uniform grid partitioning: The authors in [11] proposed a guideline in which a grid size $m=\sqrt{\frac{N\varepsilon }{c}}$ minimizes the sum of the perturbation and non-uniformity error (*N* is the number of points and *c* is a small constant). As already explained in Section 3.2, the perturbation and non-uniformity errors are the sources of the estimation error. Therefore, we generate an m×m uniform grid within the hotspots to balance these two errors and add Laplace noise to the actual count value of each grid cell. We describe the *GridPartitioning* procedure in Algorithm 2.

Algorithm 2 first determines the grid size following the guideline in [11] (Line 2). Then, each location point in a hotspot *h* is distributed over these grid cells (Line 5). Finally, Algorithm 2 adds Laplace noise $X∼Lap(\frac{1}{\varepsilon^{c}})$ to the actual count value of each grid cell (Line 7). In conclusion, SAGA adaptively lays a uniform grid on the hotspots. The adaptive grid method in [11] first lays a coarse uniform grid to capture the distribution. Accordingly, if the distribution of the dataset is skewed, the adaptive grid method creates many meaningless cells, whereas SAGA avoids this problem by detecting the hotspots. In other words, SAGA lays a uniform grid in the regions where the points might exist.   
**Algorithm 2***GridPartitioning* procedure.**Require**: a hotspot *h*, count privacy budget $\varepsilon^{c}$ **Ensure**: a hotspot with an m×m uniform grid $h_{ug}$
1:*N* ← the number of points in *h*2:Compute the grid size $m=\sqrt{\frac{N\varepsilon^{c}}{c}}$3:Construct an m×m uniform grid in *h*4:**for** point p∈h **do**5:    Increase the count value of a grid cell in which *p* falls6:**end for**7:Perturb the cell counts by adding Laplace noise $X∼Lap(\frac{1}{\varepsilon^{c}})$8:**return**$h_{ug}$

**Theorem** **7.** *Algorithm 2 achieves $\varepsilon^{c}$-differential privacy.*

**Proof.**  Because the grid cells in each hotspot are mutually exclusive, by Theorem 4, we can use the entire budget $\varepsilon^{c}$ for each grid cell. We also already know that the sensitivity of the count() function is one. According to Theorem 1, adding the Laplace noise $X∼Lap(\frac{1}{\varepsilon^{c}})$ to the actual count value of each grid cell guarantees $\varepsilon^{c}$-differential privacy. □

In Algorithm 3, we present the overall hotspot detection algorithm in detail. First, we sort the coordinate values of the location points in the spatial domain by x-values in ascending order (Line 1). A sliding window with a width $\frac{width(D)}{\sqrt{s}}$ starts from the leftmost point (Line 5). The algorithm then checks the frequency condition to determine whether the number of points in the x-value range $\left[x_{i},x_{i}+w\right)$ is greater than or equal to $\frac{\left|O\right|}{f}$ (Line 7). In the same manner, for the location points that satisfy the frequency condition, the algorithm makes another y-value coordinate list (Line 10). Subsequently, another sliding window with a height $\frac{height(D)}{\sqrt{s}}$ starts from the undermost point (Line 12). If the number of points in the overlapping region of two sliding windows is greater than or equal to $\frac{\left|O\right|}{f}$, this region is chosen as the hotspot (Line 20). If the sliding widow starts from the rightmost point or topmost point, the algorithm knows it has reached the boundary and terminates the hotspot detection procedure (Lines 25 and 29).
**Algorithm 3** SAGA procedure.**Require:** a spatial domain *D*, a set of location points *O*, size parameter *s*, frequency parameter *f*, count privacy budget $\varepsilon^{c}$, boundary privacy budget $\varepsilon^{b}$ **Ensure:** a set of hotspots *H*
 1:$L_{X}$ = SortByX(O,asc) ← a list with points in *O* sorted by x-value in ascending order 2:$w=width\left(D\right)/\sqrt{s}$ 3:$h=height\left(D\right)/\sqrt{s}$ 4:$o_{i}$ ← the first point in $L_{X}$, which has the coordinates $\left(x_{i},y_{i}\right)$ 5:**while**$o_{i}$ exists **do** 6:   $CL_{X}$ ← a list with candidate points in $L_{X}$ which have x-value in $\left[x_{i},x_{i}+w\right)$ 7:   **if** $|CL_{X}|\geq \frac{\left|O\right|}{f}$ **then** 8:     $x_{left}$ = *BoundaryGeneration*$\left(CL_{X},\varepsilon^{b}/4\right)$ 9:     $x_{right}$ = *BoundaryGeneration*$(SortByX(CL_{X}$$,desc),\varepsilon^{b}/4)$10:     $L_{Y}=$$SortByY(CL_{X},asc)$ ← a list with points in $CL_{X}$ sorted by y-value in ascending order11:     $o_{j}$ ← the first point in $L_{Y}$, which has the coordinates $\left(x_{j},y_{j}\right)$12:     **while** $o_{j}$ exists **do**13:       $CL_{Y}$ ← a list with points in $L_{Y}$, which have the y-value in $\left[y_{j},y_{j}+h\right)$14:       **if** $|CL_{Y}|\geq \frac{\left|O\right|}{f}$ **then**15:         $y_{bot}$ = *BoundaryGeneration*$\left(CL_{y},\varepsilon^{b}/4\right)$16:         $y_{top}$ = *BoundaryGeneration*(SortByY$\left(CL_{y},desc\right),\varepsilon^{b}/4)$17:         *h* ← a hotspot with the coordinates of bottom-left $\left(x_{left},y_{bot}\right)$ and top-right $\left(x_{right},y_{top}\right)$18:         **if** *h* does not overlap any hotspots in *H* **then**19:           *GridPartitioning*$\left(h,\varepsilon^{c}\right)$20:           Add *h* into *H*21:           Remove the points in $CL_{Y}$ from $L_{X}$ and $L_{Y}$22:           $o_{j}$ ← the next point in $CL_{Y}$ with a y-value greater than $y_{j}+h$23:         **end if**24:       **else**25:         $o_{j}$ ← the next point in $CL_{Y}$26:       **end if**27:     **end while**28:   **else**29:     $o_{i}$ ← the next point in $L_{X}$30:   **end if**31:**end while**32:**return***H*


**Figure 8:**
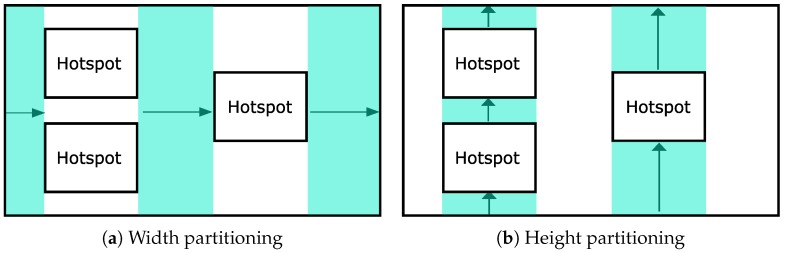
An example of the remaining regions’ partition.

Although the detected hotspots cover a part of the entire spatial domain, the sub-regions that do not have any hotspots could exist. For the sub-regions that do not contain any hotspots, we could not accurately estimate the number of objects, since the accuracy wholly depends on the distribution of the entire domain (the uniform distribution assumption). Thus, we also split the remaining regions based on the detected hotspots as illustrated in Figure 8a,b. Note that we do not need any privacy budget to formulate the boundaries of these sub-regions, since utilizing already noisy boundaries of the hotspots does not degrade the privacy guarantee. SAGA also does the *GridPartitioning* procedure in these divided sub-regions to optimize the estimation errors. However, the difference is that we can use the whole privacy budget to build a uniform grid because any privacy budget is needed to divide the remaining regions.

**Theorem** **8.** *Algorithm 3 achieves $\left(\varepsilon^{b}+\varepsilon^{c}\right)$-differential privacy.*

**Proof.**  Algorithm 3 consists of two phases: BoundaryGeneration and GridPartitioning. In the process of building a hotspot boundary, because we need four noisy coordinate values (bottom-left and top-right), the boundary privacy budget $\varepsilon^{b}$ should be divided into four pieces, that is $\varepsilon^{\tilde{b}}=\varepsilon^{b}/4$. By Theorem 3 and Theorem 6, formulating one hotspot boundary achieves $\varepsilon^{b}$-differential privacy. Furthermore, because of the exclusiveness condition (Definition 1), by Theorem 4, we can use the boundary budget $\varepsilon^{b}$ independently.

Algorithm 3 later constructs a uniform grid in each hotspot. In the same manner, the entire count privacy budget $\varepsilon^{c}$ can be used for each hotspot to build a uniform grid. Accordingly, by Theorem 4 and Theorem 7, this procedure achieves $\varepsilon^{c}$-differential privacy.

We have proven that the hotspot boundary generation guarantees $\varepsilon^{b}$-differential privacy, and the uniform grid partitioning algorithm satisfies $\varepsilon^{c}$-differential privacy. These two algorithms run on the same database as described in Algorithm 3. Therefore, by Theorem 3, Algorithm 3 achieves $\left(\varepsilon^{b}+\varepsilon^{c}\right)$-differential privacy. □

Choice of the values of parameters *s* and *f*: As shown in Section 4.2.2, to detect the hotspots, SAGA requires two parameter values of *s* and *f*. Recall that these values specify the minimum size and number of points in each hotspot, respectively. Because these values can reveal information about the underlying datasets, they must be determined privately [27].

To address the issue stated above, we set the parameter values based on the grid size $m=\sqrt{\frac{N\varepsilon }{c}}$. Suppose the total number of points in the entire spatial domain is *F*. Then, each hotspot has at least $\frac{F}{f}$ number of points following the definition of the hotspots. Because we would like to minimize the range query estimation error, we must find a set of hotspots that have a grid size of at least one ($m=\sqrt{\frac{N\varepsilon }{c}}=1$). More specifically, we can rewrite the above equation as $1=\sqrt{\frac{F\varepsilon^{c}}{fc}}$, which is equivalent to $f=\frac{F\varepsilon^{c}}{c}$.

Subsequently, we now consider the value of *s*. Intuitively, two parameters *s* and *f* together determine the relative density of the hotspots ($\frac{s}{f}$). Accordingly, if the distribution of the underlying dataset is skewed, using a higher *s* value would better capture the distribution. The choice of *s*, however, is difficult for the reason that the relative density can also reveal information of the underlying dataset. Therefore, we use s=f (the minimum relative density for the hotspots equals that of the entire spatial domain) in our implementation of SAGA and will demonstrate in the experiments that SAGA achieves reasonably good utility in this setting.

Choice of the value of parameter *c*: Recall that we set the value of $f=\frac{F\varepsilon^{c}}{c}$. At a glance, as we already know that the total number of points in the entire spatial domain is *F* and we also know the privacy budget $\varepsilon^{c}$, the value of *c* determines the minimum number of points in each hotspot. Because we add the Laplace noise to ensure the differential privacy requirement, the noisy count of each grid cell should not be overwhelmed by the Laplace noise. Therefore, we empirically use the value of c=32 in our implementation of SAGA, which also serves the balanced coordinate value range for determining the noisy hotspot boundary. This value is also used in [9,12] to define the minimum count of a leaf node in a kd-tree and h-tree.

## 5. Experimental Study

In this section, we evaluate the range query estimation accuracy of SAGA with state-of-the-art PSD methods [9,11,12,13] on four real datasets. We first explain the experiment setup and then present our experimental results.

### 5.1. Experiments Setup

**Figure 9:**
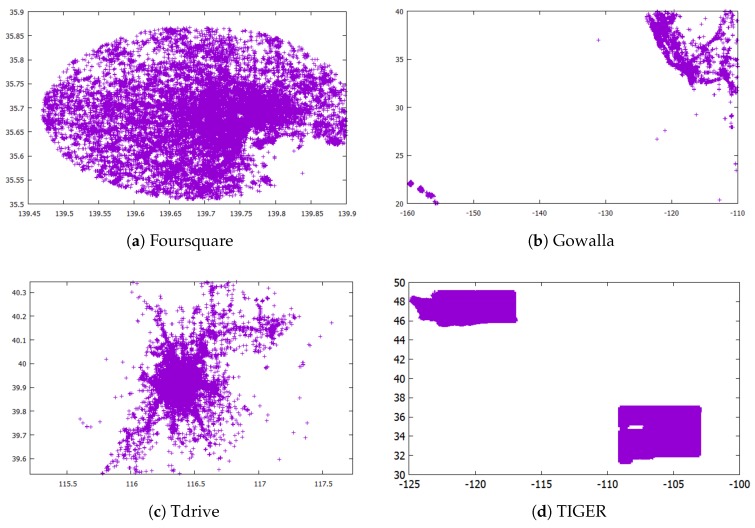
Illustration of datasets.

Datasets: We performed experiments on four real datasets illustrated in Figure 9. The information about each dataset is also described in Table 1.

The Foursquare dataset (https://sites.google.com/site/yangdingqi/home/foursquare-dataset) contains the check-ins in New York and Tokyo. In this experiment, we only use the GPS coordinates in Tokyo, which are presented in Figure 9a.

The Gowalla (https://snap.stanford.edu/data/loc-gowalla.html) dataset contains the check-ins of 6.4 M records from location-based social networking services. To make a skewed distribution, we only use part of the records located in Hawaii, shown in Figure 9b.

The Tdrive dataset (https://www.microsoft.com/en-us/research/publication/t-drive-trajectory-data-sample/) includes one-week trajectories of 10,357 taxis in Beijing. This data consist of ID, time, longitude and latitude. Here, we do not consider the time and only use the location attributes (Figure 9c).

The TIGER dataset is used in the experimental analysis of [9,11,12,13]. We obtained this dataset from 2006 TIGER/Line from the U.S. Census (https://www.census.gov/geo/maps-data/data/tiger-line.html). This dataset includes the sequential GPS coordinates of road intersections. Here, we use the GPS coordinates of the states of Washington and New Mexico (Figure 9d).

Measurement: We evaluate the accuracy of range query estimation using the average relative error. First, the relative error is measured as follows.

$$RelativeError\left(q\right)=\frac{\left|Noisy(q)-Real(q)\right|}{\max\{Real(q),\lambda \}}$$

Here, Noisy(q) means that the count estimated with PSD, and Real(q) represents the actual number of points in the range query. λ is a factor that is used when the real count is too small. This factor mitigates the influences of small counts on the relative error. Furthermore, this factor also serves the purpose of limiting division by zero. We set λ as 0.001×|D|, where |D| is the total number of points in dataset *D*. For the set of range queries *Q*, the average relative error is calculated as follows.

$$AverageRelativeError\left(Q\right)=\frac{\sum_{q\in Q}RelativeError\left(q\right)}{\left|Q\right|}$$

Queries: We use the three types of range queries of sizes 0.1%(large), 0.01%(medium) and 0.001%(small) from the dataset domain. These range queries have a square shape. To measure the average relative error, we randomly generate 10,000 queries for each dataset.

Methods: We evaluate SAGA with five PSD methods: Uniform Grid (UG) [11], Adaptive Grid (AG) [11], KD-Tree (KDT) [9], H-Tree (HT) [12], PrivTree (PT) [13]. UG lays m×m grid cells of equal size on the spatial domain and adds the Laplace noise in each count value of a grid cell. AG first lays a coarse uniform grid. Then, based on the noisy count of each grid cell, AG lays another uniform grid. In the case of KDT, because KDT has several variants, we use a kd-hybrid method that demonstrates the best performance [9]. The kd-hybrid method first generates a kd-tree, and after the switching height, a quadtree is built. HT is based on the equi-depth histogram of size m×m, which gives a similar number of points in each sub-region. PT is the state-of-the-art PSD method that is based on the quadtree. PT splits each sub-region by using the biased count value, thereby eliminating the need for the predefined total height.

Parameters: Next, we describe the parameters used in this experiment. Table 2 describes detailed parameter configurations for the five PSD methods. The size and frequency parameter values of SAGA for each dataset are determined according to our discussion in Section 4.2.3 and described in Table 3. The value of *c* is set to 10 for UG and AG and three for HT, following the analysis in [11,12]. For KDT, because we use the kd-hybrid method, we set the total height to six and the switching height to three, which is half of the total height. PT speculates about the data distribution based on the biased count that is calculated by the parameter δ. δ serves the purpose of having a balance between the amount of bias and noise. Here, we use $\delta =\frac{7}{3}\cdot \frac{1}{\alpha \varepsilon }\cdot \ln\left(4\right)$ following the analysis in [13]. For all the data-dependent methods including SAGA, we must divide the total privacy budget. We assign 40% of the total privacy budget for building the structures of the histogram and the remainder for adding the Laplace noise. Among the data-independent methods, AG and PT must divide the total privacy budget: AG creates two-level grids, and PT uses a part of the budget to build a quadtree structure and the remainder to add the Laplace noise at the leaf node count. Accordingly, we equally divide the total privacy budget for AG and PT.

### 5.2. Experiment Results

**Figure 10:**
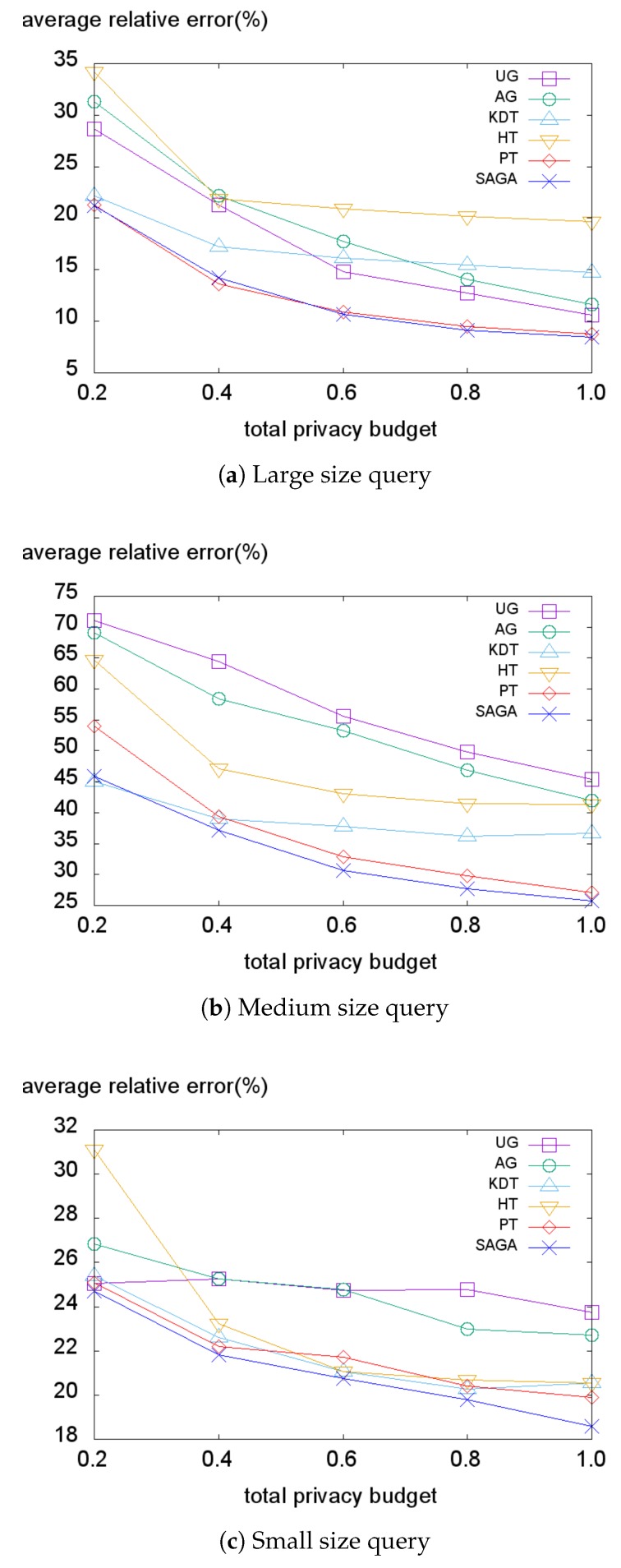
Average relative error on the Foursquare dataset.

**Figure 11:**
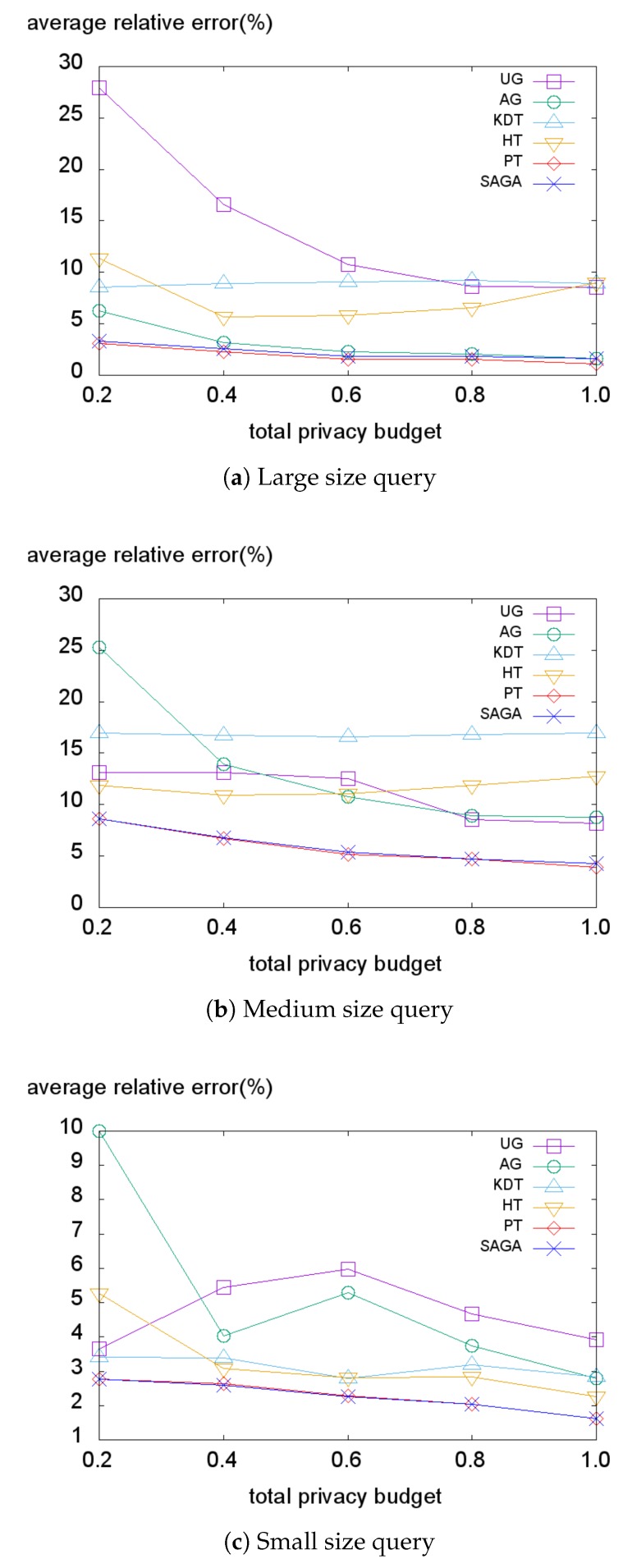
Average relative error on the Gowalla dataset.

**Figure 12:**
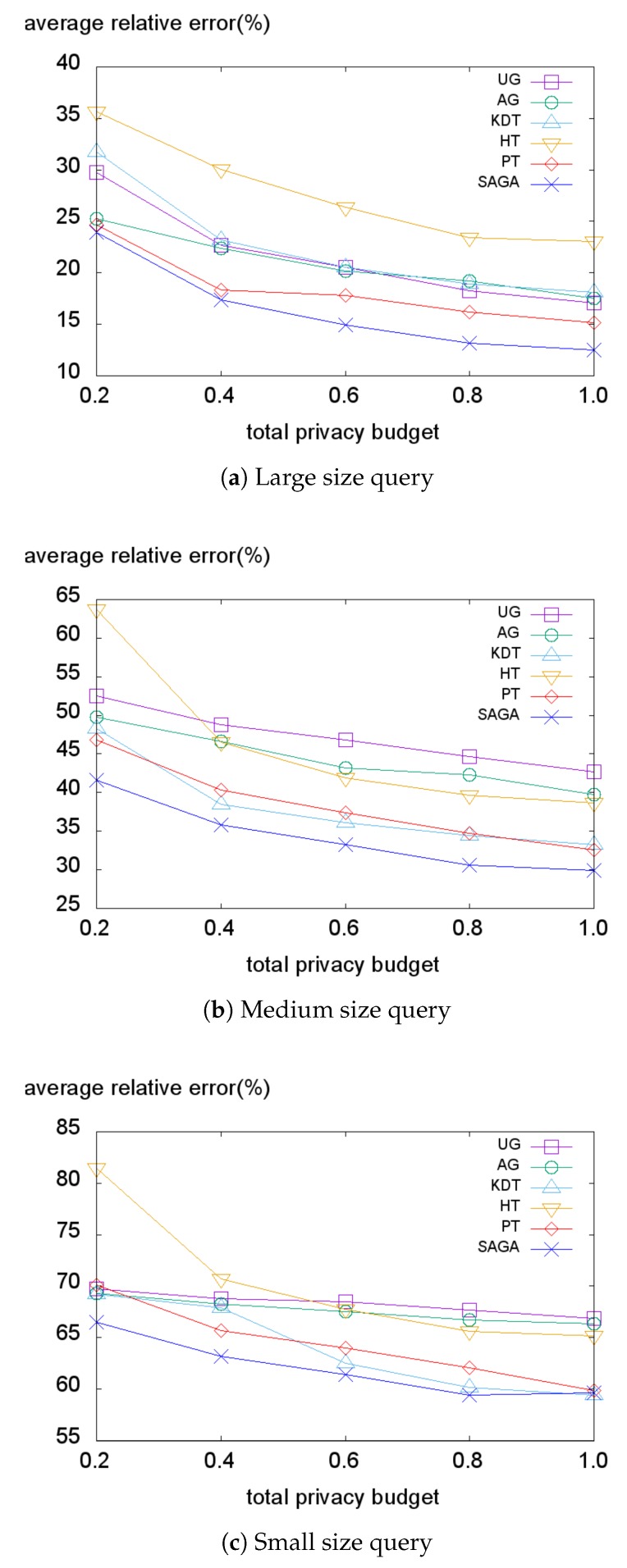
Average relative error on the Tdrive dataset.

**Figure 13:**
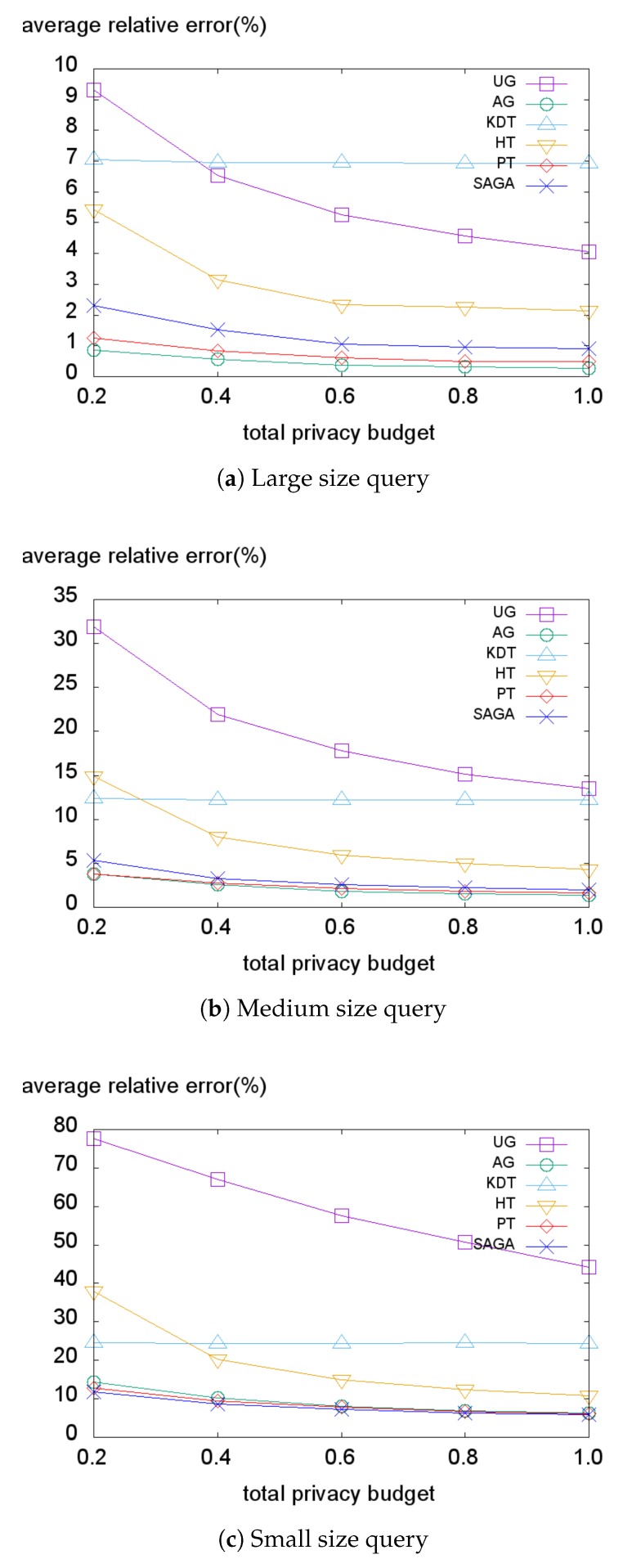
Average relative error on the TIGER dataset.

Figure 10, Figure 11, Figure 12 and Figure 13 illustrate the average relative error on each dataset while varying the total privacy budget. As expected, the overall average relative error tends to decrease when the total budget grows. The reason is that the variance of the Laplace noise decreases for all PSD methods and the structures of the PSD become similar to the data distribution for the data-dependent PSD methods.

We first evaluate the error on the Foursquare dataset represented in Figure 10. When the size of the query is large, the data-independent methods (UG, AG, PT) tend to achieve lower average relative errors than the data-dependent methods (Figure 10a). This is because when the size of the query is large, the number of fully-covered sub-regions that only cause the perturbation error increases. Further, because the Foursquare dataset is a high-density dataset, the non-uniformity error has little effect on the average relative error, resulting in better performance by data-independent methods. However, when the query size becomes smaller, the portion of the non-uniformity error within the average relative error increases. Therefore, the data-dependent methods (KDT, HT, SAGA) tend to produce lesser errors than the data-independent methods when smaller size queries are applied (Figure 10b,c). This indicates that the data-independent methods are vulnerable to the non-uniformity error and demonstrates that SAGA effectively reduces the non-uniformity error by taking advantage of the hotspots.

Next, we investigate the average relative error on the *Gowalla* dataset illustrated in Figure 11. Although this dataset has a very skewed distribution, the data-independent methods (AG, PT) perform well in large size query conditions (Figure 11a). However, the data-dependent methods provide lower errors in small size query conditions (Figure 11c). This also reveals that the smaller size query produces a higher non-uniformity error. Surprisingly, the state-of-the-art method PT consistently demonstrates almost equivalent errors compared to SAGA. Even though PT is a data-dependent method, it is tolerant of the non-uniformity error based on the biased counts.

Subsequently, we evaluate our method on the Tdrive dataset (Figure 12). Here, in all cases, SAGA consistently performs best. As explained in Section 5.1, this dataset contains the trajectories of taxis in Beijing. Therefore, this dataset has a typical population distribution of a metropolitan area where most of the people exist in the center of the city. Accordingly, the Tdrive dataset has a more skewed distribution than the Foursquare dataset. Overall, SAGA significantly outperforms other methods on the Tdrive dataset because the hotspots enable the SAGA histogram to handle the skewness of the dataset and reduce the non-uniformity error. Although PT and AG adapt to the data distribution well enough, they still suffer from the skewness problem with the existence of some outliers.

Finally, we evaluate the error on the TIGER dataset represented in Figure 13. When using the large size query (Figure 13a), two data-independent methods (AG, PT) demonstrate superior performance, followed by SAGA. This also indicates that the data-independent methods can efficiently handle the perturbation error. However, in medium and small size query settings, the performance differences between the data-independent methods (AG, PT) and the data-dependent methods (KDT, HT, SAGA) degrade. Even in the case of a small size query setting, SAGA demonstrates slightly better performance than AG and PT. We conclude that adaptively laying the uniform grid based on the optimal grid size enables SAGA to handle the perturbation error. Accordingly, SAGA demonstrates comparably good performance even when the dataset is not too skewed.

In summary, the data-dependent methods work well when the dataset has a skewed distribution, and the portion of non-uniformity error is larger than the perturbation error. In particular, SAGA consistently demonstrates better performance than other data-dependent methods (KDT, HT). Further, SAGA also achieves competitive utility compared to the data-independent methods with moderately distributed datasets.

## 6. Conclusions

Mobile crowdsensing is an attractive paradigm that enables numerous large-scale sensing applications through the prevalent mobile devices. In this paper, we explored the privacy issue associated with mobile crowdsensing. To protect the location privacy of the workers involved in mobile crowdsensing, we present a novel PSD method based on hotspots. We observed that existing methods provide relatively poor performance because they pay little attention to the domain size of the input dataset. To overcome this problem, we propose a skew-aware grid partitioning method named SAGA. SAGA leverages the notion of the hotspot, which has high relative density. We further optimize the errors by laying a uniform grid in each hotspot. By combining the hotspots and uniform grid, we divide the entire spatial domain into a set of more skew-tolerant sub-regions. We conducted several experiments on four real datasets with state-of-the-art PSD methods. Experimental results demonstrate that SAGA provides better utility than existing PSD approaches.

## Figures and Tables

**Table 1:** Information about datasets.

Dataset	Total Number of Points	Domain Size
*Foursquare*	61,391	$0.5{}^{\circ }\times 0.4{}^{\circ }$
*Gowalla*	132,088	$60{}^{\circ }\times 20{}^{\circ }$
*Tdrive*	28,532	$1{}^{\circ }\times 0.8{}^{\circ }$
*TIGER*	1,325,737	$30{}^{\circ }\times 20{}^{\circ }$

**Table 2:** Parameter configurations for each method.

Method	Description
**UG**	Grid size: $m=\sqrt{\frac{N\varepsilon }{c}}$
**AG**	First grid size: $m_{1}=\max\left\{10,\frac{1}{4}\sqrt{\frac{N\alpha \varepsilon }{c}}\right\}$, Second grid size: $m_{2}=\sqrt{\frac{N^{{}^{\prime }}\left(1-\alpha \right)\varepsilon }{c}}$
**KDT**	Total height: 6, Switching height: 3
**HT**	h-tree size: $m=\sqrt{\frac{N\varepsilon }{c}}$
**PT**	Bias factor: $\delta =\frac{7}{3}\cdot \frac{1}{\varepsilon }\cdot \ln\left(4\right)$

**Table 3:** *s* and *f* values of SAGA used in each dataset (s=f).

Dataset	Total privacy budget				
	ε=0.2	ε=0.4	ε=0.6	ε=0.8	ε=1.0
*Foursquare*	230	460	690	920	1151
*Gowalla*	495	990	1486	1981	2476
*Tdrive*	107	214	321	428	535
*TIGER*	4971	9943	14,914	19,886	24,857

## Abbreviations

The following abbreviations are used in this manuscript:

DP	Differential Privacy
PSD	Private Spatial Decompositions
SAGA	Skew-Aware Grid pArtitioning
